# Cardiac tamponade, a rare complication of gastric cardia cancer resection after neoadjuvant chemotherapy combined with immunotherapy: a case report and literature review

**DOI:** 10.3389/fonc.2023.1189500

**Published:** 2023-08-22

**Authors:** Wei Du, Hemei Wang, Junmei Shen, Xi Qiao, Jifang Yao, Chao Li

**Affiliations:** ^1^ Department of Anesthesiology, The Fourth Hospital of Hebei Medical University, Shijiazhuang, Hebei, China; ^2^ Department of Thoracic Surgery, The Fourth Hospital of Hebei Medical University, Shijiazhuang, Hebei, China

**Keywords:** cardiac tamponade, gastric cardia cancer, immunotherapy, neoadjuvant chemotherapy, transthoracic gastric resection

## Abstract

Transthoracic cardia resection is a technically well-established surgical procedure. However, acute cardiac tamponade in the early postoperative period is extremely rare. The occurrence is life-threatening to the patient. It also poses a great clinical challenge for perioperative management. To date, few cases of pericardial tamponade have been reported in gastric cancer resection performed after neoadjuvant chemotherapy combined with immunotherapy. We present the case of a 62-year-old woman who received neoadjuvant chemotherapy combined with immunotherapy before surgery, followed by transthoracic surgery. A life-threatening complication, pericardial tamponade, occurred in the early postoperative period. The successful outcome was achieved in through multidisciplinary collaboration.

## Introduction

The global incidence and mortality of gastric cancer have shown a decreasing trend over the past decades (1). Nevertheless, the incidence of gastric cancer is still high in some countries, especially in Japan and China (1). The main population of gastric cancer is advanced stage patients, and surgical treatment is an important strategy to improve the survival rate (2). Numerous studies have confirmed that neoadjuvant chemotherapy shows good application prospects and has become an important component in the multidisciplinary comprehensive treatment of malignant tumors (3–6). With the great progress in immunotherapy and the accumulation of relevant clinical evidence, significant changes have occurred in the field of cancer treatment. Some studies have confirmed that the combination of immunotherapy on the basis of neoadjuvant chemotherapy significantly improves the survival benefit of patients with adenocarcinoma of the esophagogastric junction (7).

In general, transthoracic cardia cancer resection is a technically mature surgical procedure. The postoperative period after transthoracic cardia cancer resection is associated with many complications, such as pulmonary complications such as pneumonia, pulmonary atelectasis, pleural effusion; cardiac arrhythmias and even digestive fistulae (8). However, the occurrence of acute cardiac tamponade in the early postoperative period is extremely rare and simultaneously poses a great clinical challenge for perioperative management. We describe a case of a 62-year-old woman with pericardial tamponade, who underwent transthoracic cardia cancer resection after neoadjuvant chemotherapy combined with immunotherapy. Written informed consent was obtained before publication of this report.

## Case report

A 62-year-old woman has been suffering from upper abdominal pain for six months. Gastroscopy revealed an esophagogastric junction lesion, and subsequent pathology confirmed the diagnosis of cardia Signet-ring cell carcinoma (H22-07643). Thoracic and abdominal CT scan revealed thickening of the cardia wall, which was considered malignant. Multiple small hemangiomas of the liver. Left lung upper lobe calcified spots. Clinical stage cT3N1M0.

**Figure 1:**
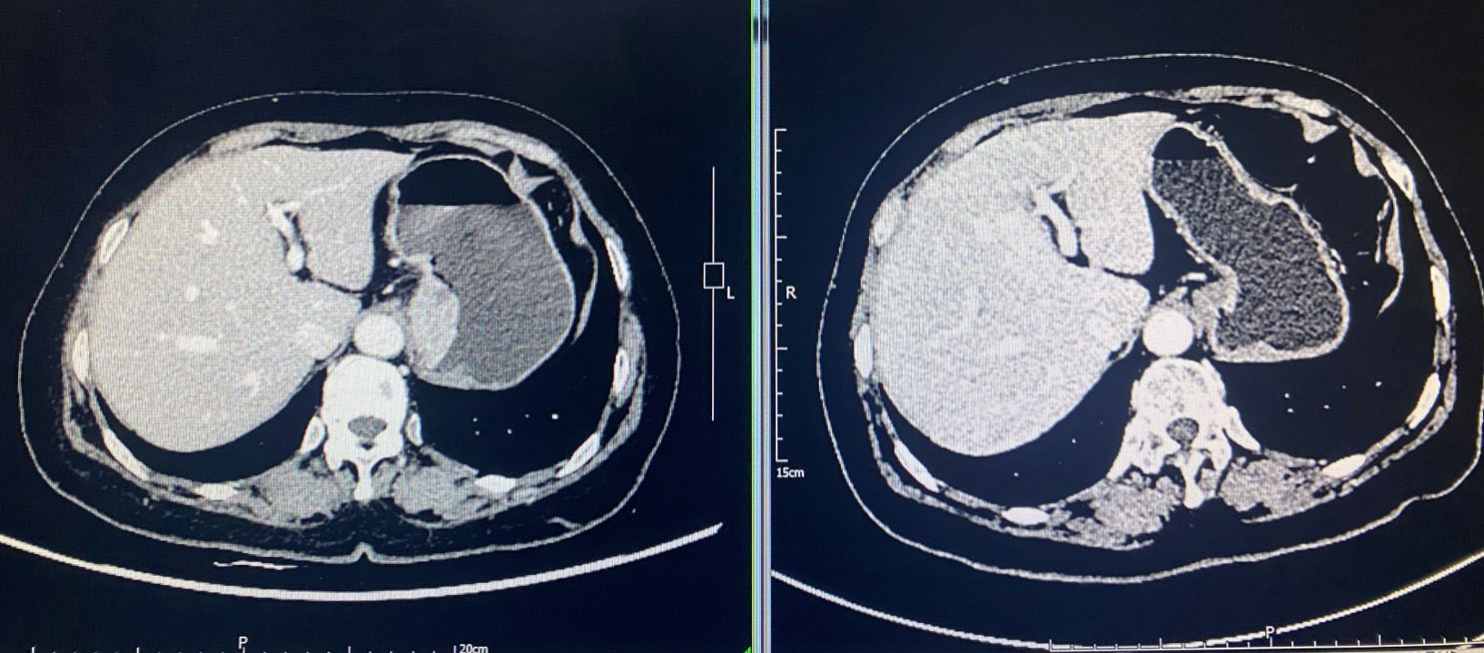
Comparison of abdominal CT scan before and after neoadjuvant therapy. Tumor achieved partial response (PR) with a 50% reduction in the sum of the largest diameters of the tumor target lesions.

After all the examinations were completed, neoadjuvant chemotherapy combined with immunotherapy was performed. The chemotherapy program XELOX (injection oxaliplatin (T) 200mg + capecitabine 1.5g 2/day d1-d14), and at the same time the patient administrated with tirilizumab as immunotherapy. The process was smooth and there was no obvious adverse reaction. One month after two cycles of neoadjuvant chemotherapy combined with immunotherapy, chest and abdominal CT scan was rechecked, and the tumor was significantly shrank compared with the preoperative CT. The tumor lesion achieved partial response (PR) and the sum of the largest diameters of the target lesions was reduced by about 50% (**Figure 1**). After preoperative examination, there was no relevant contraindication. Left-sided transthoracic and gastric cardiac cancer resection and esophagogastrostomy were performed. The surgically resected tumor, which is approximately 5×4×1 cm in size, is located on the small curvature side of the gastric cardia. Tumor and lymph nodes unrelated to the pericardium. Intraoperatively, a rib spreader was used in order to ensure surgical visualization. The heart may be slightly squeezed during chest-opening surgery, but hemodynamic stability is achieved. The operation was successfully completed. The patient is then returned to the ward.

48 hours after surgery, the patient presented with sudden onset of panic, chest tightness, tachycardia, and hypotension. There is no evidence of bleeding, and a minor amount of pleural effusion is present on both sides of the left chest as a result of the operation. Blood gas analysis is normal. Although myocardial ischemia was suspected, the emergency ECG revealed low voltage in the limb leads and an aberrant T wave in the anterior wall leads. The critical cardiac troponin I (cTnI) concentration was 1.4 μg/L. Echocardiography showed a small to moderate amount of pericardial effusion. Measurement of 19 cmH_2_O central venous pressure is required. The patient’s persistent hypotension was treated with intravenous injection of norepinephrine. While preparing for ultrasound-guided pericardiocentesis, the patient stated that her extremity pain and chest tightness were worsening. Then sudden loss of consciousness. Cardiopulmonary cerebral resuscitation and tracheal intubation were performed immediately. After pericardiocentesis, the patient was inserted into the pericardial drainage tube and recovery of spontaneous rhythm occurred within 9 minutes. Drainage fluid of dark red blood totally 460ml was withdrawn. Despite improvement in the patient’s hemodynamics and awareness, blood pressure remains low. Three hours later, echocardiography revealed heterogenous hypoechogenicity of the pericardium. The left ventricular posterior wall was 2.1cm thick, the left ventricular lateral wall was 1.2cm thick and the thickness of the apical part was 1.1cm. The movement of the left ventricular was limited. Thoracotomy was then carried out immediately.

**Figure 2:**
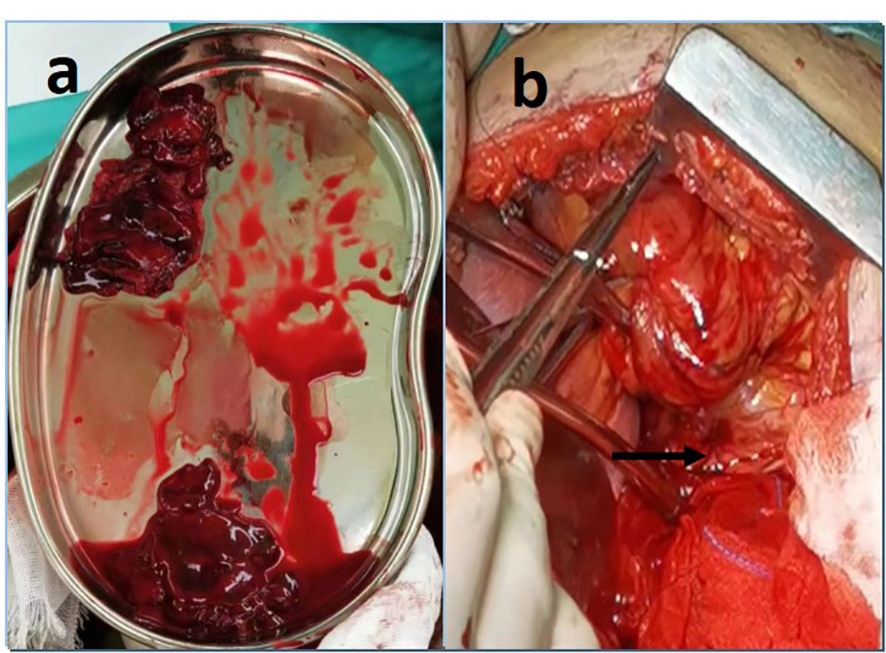
**(A)** After incised the pericardium, approximately 60ml of blood clot was removed. **(B)** A small blood vessel on the lateral wall of the left ventricle, with angiomatous changes and active bleeding.

With a patient’s heart rate of 125 bpm and an arterial pressure of 70/50mmHg. The tripartite staff is fully prepared. In an attempt to ensure hemodynamic stability during anesthesia. Once general anesthesia has been successfully achieved, assume the right recumbent position and enter the thoracic cavity through the original incision. Notice that the pericardium is complete and smooth, and that there is no bleeding spot on the surface. The pericardium is incised approximately 6cm behind the phrenic nerve, and dark red blood clots of approximately 60ml. (**Figure 2A**) A small blood vessel on the myocardial surface can be observed on the lateral wall of the left ventricle, with angiomatous changes and active hemorrhage (**Figure 2B**).

Using 4/0 prolene with a knitted “U” joint for stitching. Patients’ hemodynamics were subsequently improved. Transferred to the ICU for further treatment postoperatively.

Postoperative cTnI, BNP, and other biomarkers of myocardial injury progressively decreased. Hemodynamics gradually stabilized. Postoperative pathology(22-35953): Signet-ring cell carcinoma, tumor regression Grade 2, ypT1bN0M0. Follow-up to the current 9 months is good. Further chemotherapy was performed last month, and the process was successful.

## Discussion

Cardiac tamponade is a symptom of cardiac compression due to slow or acute accumulation of fluid, pus, blood, clot or gas in the pericardium (9), and increased intrapericardial pressure causes impaired diastolic filling function and decreased cardiac output. Once it occurs the condition is aggressive and life-threatening (9). Therefore it is essential to identify the etiology and to make rapid diagnosis and treatment of the disease.

## The etiology

The etiology of acute pericardial tamponade includes inflammatory and non-inflammatory factors (10). Causes of acute pericarditis include bacterial and Mycobacterium tuberculosis infection, connective tissue disease, and uremia. Acute pericardial hemorrhage: ventricular wall rupture, aortic aneurysm or entrapment rupture into the pericardium, chest wall trauma, and cancer metastasis.

After surgery, the etiology of acute pericardial tamponade in this patient was analyzed as acute pericardial effusion. After excluding ventricular wall rupture and aortic dissection. It seemed that the possible causes were pericardial metastasis of gastric cancer and/or trauma from the first thoracic surgery.

Cases of advanced gastric cancer combined with pericardial metastasis presenting with massive pericardial effusion leading to pericardial tamponade are relatively uncommon, but still intermittently reported. By entering a search formula in the Web of Science Core Collection database (WoSCC): TS=(pericardial effusion OR cardiac tamponade) AND TS=(gastric carcinoma OR gastric cancer OR stomach carcinoma OR stomach cancer OR stomach neoplasm*) AND TS=(chemotherapy). The majority are case reports. **Table 1** lists 6 case reports of gastric cancer metastasis to the pericardium presenting with pericardial effusion or pericardial tamponade (11–16). However, was this patient combined with pericardial metastases? Pericardial metastasis could be basically excluded by the following reasons: 1) No pericardial metastasis indicated by CT scan before the first surgery; 2) no pericardial metastasis was detected by the surgeon’s exploration during the first transthoracic surgery; 3) no metastasis on exploration after incision of the pericardium in the second surgery. The reason cannot be ruled out as a result of trauma from the first chest surgery. Due to the fact that the heart may be slightly squeezed during the chest surgery. But rarely such serious complication of postoperative pericardial tamponade occurs. Yoshiaki Mizuguchi et al. reported a rare case of pericardial tamponade after resection of esophageal cancer (17). However, the case mentioned above did not receive preoperative application of neoadjuvant chemotherapy and immunotherapy. Many biological agents such as tumor necrosis factor (TNF) inhibitors (18), rituximab (19), tolizumab (20), and immune checkpoint inhibitors (21) may induce small-vessel vasculitis and drug-induced vasculitis, which increases the fragility of the small vessels and makes them more vulnerable to damage. Based on this, there is some possibility that the patient in this case also developed small vessel vasculitis, which was not attended to preoperatively.

**Table 1:** Reported cases of gastric cancer metastasis to the pericardium presenting with pericardial effusion or pericardial tamponade.

	Age	Gender	Clinical presentation	Diagnosis	Management	Survival
**Varvarigos, N et al., 2001 (** 11 **)**	79	M	progressive dyspnea, cough, and edema of both legs	gastric cancer; heart failure.	Pericardiocentesis; chemotherapeutic sessions with platine, 5-fluorouracil (5-FU), and mitomycin and surgical operation (total gastrectomy)	>18 months
**Funk, L et al., 2003 (** 12 **)**	78	F	palpitation, tachycardia and progressive dyspnea	gastric cancer	pericardial drainage; patient suffered stroke and the chemotherapy could not be initiated.	2 months
**Wiedmann, A et al., 2005 (** 13 **)**	50	M	dysphagia, dyspnea, tachycardia, and hypotension	esophageal carcinoma with lung and liver metastases	antibiotics, repeated pleurocentesis and pericardial drainage	3 months
**Baba, Yoshifumi et al., 2007 (** 14 **)**	53	F	lower abdominal mass	bilateral ovarian tumor;Krukenberg tumor and pericardial metastasis	total gastrectomy with radical lymph node dissection and bilateral ovarian resection;Adjuvant chemotherapy with irinotecan (CPT-11) and low-dose cisplatin (CDDP)	13 months
**Kusaba, Hitoshi et al., 2008 (** 15 **)**	59	M	dyspnea, anterior chest oppression, and hypotension	gastric cancer	pericardiocentesis followed by systemic chemotherapy consisting of TS-1 and cisplatin (CDDP)	>5 months
**Zhang, Bi-li et al., 2010 (** 16 **)**	56	F	dyspnea, anterior chest oppression, and hypotension	gastric cancer;cardiac tamponade	percutaneous pericardiocentesis followed by systemic chemotherapy (oxaliplatin and sequential 5-fluorouracil plus leucovorin).	>6months

## Rapid bedside assessment and differential diagnosis

The use of the “SHOCK” memory at the bedside helps to promptly recognize obstructive shock from cardiac tamponade and to exclude other etiologies of shock (22). Septic shock (S) or distributive shock is distinguished from other forms of shock by high cardiac output. Hypovolemic shock (H) with low filling pressures (central venous pressure [CVP], history of volume loss). However, obstructive shock (O) and cardiogenic shock (C) combine with high cardiac filling pressures. Obstructive shock has clear lung fields on physical examination and chest radiography. Pneumothorax, pulmonary embolism, and cardiac tamponade are common causes of obstructive shock, so the next pass through the physical examination focuses on differentiating between these causes. The possibility of cardiac tamponade in patients who are hypotensive or hemodynamically unstable is quickly identified by this method combinations/other kinds of shock(K) (23).

In patients with suspected pericardial tamponade, cardiac ultrasound is the diagnostic method of choice and should be performed immediately. CT and MRI are not routinely performed in patients with suspected pericardial tamponade, but are useful in excluding possible mediastinal or pulmonary accompaniments in patients with large pericardial effusions.

## Treatment programs

Pericardial drainage is feasible if the patient has a confirmed diagnosis of pericardial tamponade (10). Pericardial drainage should be done as soon as possible after diagnosis if the patient is hemodynamically stable after obtaining laboratory results such as blood volume.

Indications for emergency pericardial tamponade include: pericardial effusions caused by type A aortic dissection, rupture of the ventricular wall in acute infarction, or trauma, infected septic pericardial effusions, and encapsulated effusions that cannot be treated transcutaneously (10).

## Perioperative management of patients with pericardial tamponade

Preoperative management: pericardial fluid should be drained slowly in patients with pericardial effusion to avoid pericardial decompression syndrome (9). Hypotensive and hypovolemic patients on whom vasoactive drugs are applied are given gentle intravenous fluids and blood products are applied promptly.

Induction of general anesthesia in patients with pericardial tamponade is extremely risky. Loss of sympathetic tone during induction and initiation of positive pressure ventilation. It may lead to systemic vasodilation, decreased preload, direct myocardial depression induction by anesthetic drugs and hemodynamic failure (24). Therefore avoid vasodilators, myocardial depressants, and positive pressure ventilation with large tidal volumes before uncuffing. In severely compromised individuals, it is prudent to ensure that the surgeon is gowned and gloved before induction and that the patient is prepared and covered.

Intraoperative anesthetic management focuses on maintaining hemodynamic stability (24). Key points of anesthetic management include: maintenance of cardiac output, fluid administration (adequate preload to improve right ventricular filling), maintenance of vascular tone (use of phenylephrine, vasopressin to maintain peripheral perfusion), enhances myocardial contractility (epinephrine, norepinephrine), and respiration: high-frequency, small tidal volume ventilation to avoid high peak airway pressures.

## How to prevent complications of pericardial tamponade?

Although pericardial tamponade after non-cardiac surgery is extremely rare, the consequences are devastating when it occurs. It is very important to prevent the occurrence of pericardial tamponade.1) At present, neoadjuvant chemotherapy combined with immunotherapy is used in many cases of esophagogastric cancer or esophageal cancer. In the perioperative period, we should be concerned not only about the myocardial injury of cytotoxic drugs, but also its effect on small blood vessels, which shouldn’t be ignored.2) For patients who receive neoadjuvant chemotherapy and then undergo surgery, the surgical operation should be more gentle and precise.

In conclusion, the occurrence of pericardial tamponade after transthoracic cardia cancer resection is extremely uncommon, but it is also an important cause and complication that leads to hemodynamic instability of patients after operation. Once acute pericardial tamponade occurs, it should be diagnosed and drained by puncture as soon as possible. Early surgical rescue should be performed if there are surgical indications.

## Data availability statement

The original contributions presented in the study are included in the article/supplementary material. Further inquiries can be directed to the corresponding author.

## Ethics statement

Written informed consent was obtained from the individual(s) for the publication of any potentially identifiable images or data included in this article.

## Author contributions

WD: Conceptualization, Writing – Original Draft, Methodology. HW: Methodology, Writing – Review & Editing. JS: Resources, Writing – Review & Editing. XQ: Visualization, Writing – Review & Editing. JY: Writing - Review & Editing, Resources. CL: Conceptualization, Resources, Writing – Review & Editing, Supervision.
