# Corrigendum: Cardiac tamponade, a rare complication of gastric cardia cancer resection after neoadjuvant chemotherapy combined with immunotherapy: a case report and literature review

**DOI:** 10.3389/fonc.2023.1355675

**Published:** 2024-01-04

**Authors:** Wei Du, Hemei Wang, Junmei Shen, Xi Qiao, Jifang Yao, Chao Li

**Affiliations:** ^1^ Department of Anesthesiology, The Fourth Hospital of Hebei Medical University, Shijiazhuang, Hebei, China; ^2^ Department of Thoracic Surgery, The Fourth Hospital of Hebei Medical University, Shijiazhuang, Hebei, China

**Keywords:** cardiac tamponade, gastric cardia cancer, immunotherapy, neoadjuvant chemotherapy, transthoracic gastric resection

In the published article, there was a mistake in the Funding statement. The funding statement previously stated “This work was supported by the Key Projects of Medical Science Research in Hebei Province (2023079)”. The correct statement is “**This work was supported by the Key Projects of Medical Science Research of Hebei Province (20230795)**.’’

The authors apologize for this error and state that this does not change the scientific conclusions of the article in any way. The original article has been updated.

